# Impact of rural-urban environment on metabolic profile and response to a 5-day high-fat diet

**DOI:** 10.1038/s41598-018-25092-6

**Published:** 2018-05-25

**Authors:** Dicky L. Tahapary, Karin de Ruiter, Farid Kurniawan, Yenny Djuardi, Yanan Wang, Siti M. E. Nurdin, Elisa Iskandar, Dominggus Minggu, Em Yunir, Bruno Guigas, Taniawati Supali, Patrick C. N. Rensen, Erliyani Sartono, Pradana Soewondo, Dante S. Harbuwono, Johannes W. A. Smit, Maria Yazdanbakhsh

**Affiliations:** 1 Department of Internal Medicine, Division of Endocrinology, Dr. Cipto Mangunkusumo National General Hospital, Faculty of Medicine Universitas Indonesia, Jakarta, Indonesia; 2 Department of Parasitology, Leiden University Medical Center, Leiden, The Netherlands; 3 Nangapanda Community Research Cluster, The Indonesian Medical Education and Research Institute, Universitas Indonesia, Jakarta, Indonesia; 4 Metabolic, Cardiovascular and Aging Research Cluster, The Indonesian Medical Education and Research Institute, Universitas Indonesia, Jakarta, Indonesia; 5 Department of Parasitology, Faculty of Medicine Universitas Indonesia, Jakarta, Indonesia; 6 Department of Medicine, Division of Endocrinology, Leiden University Medical Center, Leiden, The Netherlands; 7 Laboratory Unit, South East Asian Minister of Education Organization Regional Centre For Food And Nutrition, Jakarta, Indonesia; 8 Dr. W.Z. Johannes Hospital, Kupang, Indonesia; 9 Department of Internal Medicine, Radboud University Medical Centre, Nijmegen, The Netherlands

## Abstract

Epidemiological studies have indicated that rural living might be protective against type 2 diabetes development. We compared the metabolic profile and response to a short-term high-fat high-calorie diet (HFD) of men with the same genetic background living in an urban and rural area of Indonesia. First, we recruited 154 Floresian male subjects (18–65 years old), of whom 105 lived in a rural area (Flores) and 49 had migrated and lived in urban area (Jakarta) for more than 1 year. The urban group had significantly higher whole-body insulin resistance (IR), as assessed by homeostatic-model-assessment of IR (HOMA-IR), [mean difference (95% CI), p-value: 0.10 (0.02–0.17), p = 0.01]. Next, we recruited 17 urban and 17 rural age-and-BMI-matched healthy-young-male volunteers for a 5-day HFD challenge. The HOMA-IR increased in both groups similarly −0.77 (−2.03–0.49), p = 0.22]. Neither rural living nor factors associated with rural living, such as current helminth infection or total IgE, were associated with protection against acute induction of IR by HFD.

## Introduction

The prevalence of obesity and type 2 diabetes (T2D) is increasing worldwide, especially in low and middle-income countries (LMIC) that are currently facing rapid rates of urbanization^1,2^. Rural-to-urban migration has indeed been shown to be associated with increased obesity and other cardiovascular (CV) risk factors, such as dyslipidemia and hypertension^3–11^, suggesting that living in rural environment might be protective against the development of T2D.

In addition to changes towards a sedentary lifestyle and an increased dietary fat intake, migration to an urban environment is also associated with a reduced exposure to microorganisms and parasites, such as helminth infections, which are still endemic in many rural areas of LMIC^12^. Recent data suggests that helminth infections might confer a protection against the development of obesity and T2D^13–16^, presumably by promoting type-2 and regulatory immune responses and subsequent reduction in systemic inflammation^17–19^. However, it is worth mentioning that the relative contribution of helminth infections in comparison to the more established factors such as a sedentary lifestyle and diet remains to be clarified.

Urban subjects have been reported to have longer sedentary periods and shorter active periods compared to those living in rural areas^20^. Furthermore, an increase in dietary fat intake, commonly observed upon rural-to-urban migration^7,20^, has been reported to be associated with impaired insulin resistance (IR) and glucose homeostasis^21^. Mice on high-fat diet (HFD) have provided models to study obesity and the development of IR^22,23^. Similarly, in humans, short-term HFD has been utilized to study the susceptibility to the development of IR^24–28^. Using this model, it has been possible to show how risk of IR is dependent on whether the participant is Caucasian or South Asian^25,28^. Short-term HFD has also been shown to induce organ-specific and systemic inflammation as evidenced by the increase in plasma cholesteryl ester transfer protein (CETP) levels^24,29^, predominantly produced by Kupffer cells (KC)^30^, and plasma C-reactive protein (CRP) levels^24^.

Taken together, the chronic increase of energy rich diet, in addition to a more sedentary lifestyle, among people who migrate from a rural to urban areas^20^, might lead to the development of IR and T2D. However, there is still incomplete insight into the pathophysiology of the development of IR and T2D in rural-to-urban migration. In addition, there has been no study comparing the metabolic response towards a short-term HFD in terms of changes in glucose homeostasis and inflammation, between people living in urban and rural areas.

As some metabolic differences between individuals living in rural and urban area can be due to genetic differences, this study compared the metabolic profile between individuals with the same genetic background living in urban and rural areas. We also compared the metabolic and inflammatory response of individuals living in a rural and an urban area to a 5-day high-fat high-calorie (HFD) diet. Furthermore, since rural areas often go hand in hand with helminth infections and its associated IgE responses, we aimed to assess their contribution to metabolic profile. We hypothesized that individuals living in rural area would have a better metabolic profile and would be relatively more protected from the induction of IR and inflammation by the HFD compared to those living in an urban area.

## Results

### The metabolic profile of urban and rural study participants

**Figure 1:**
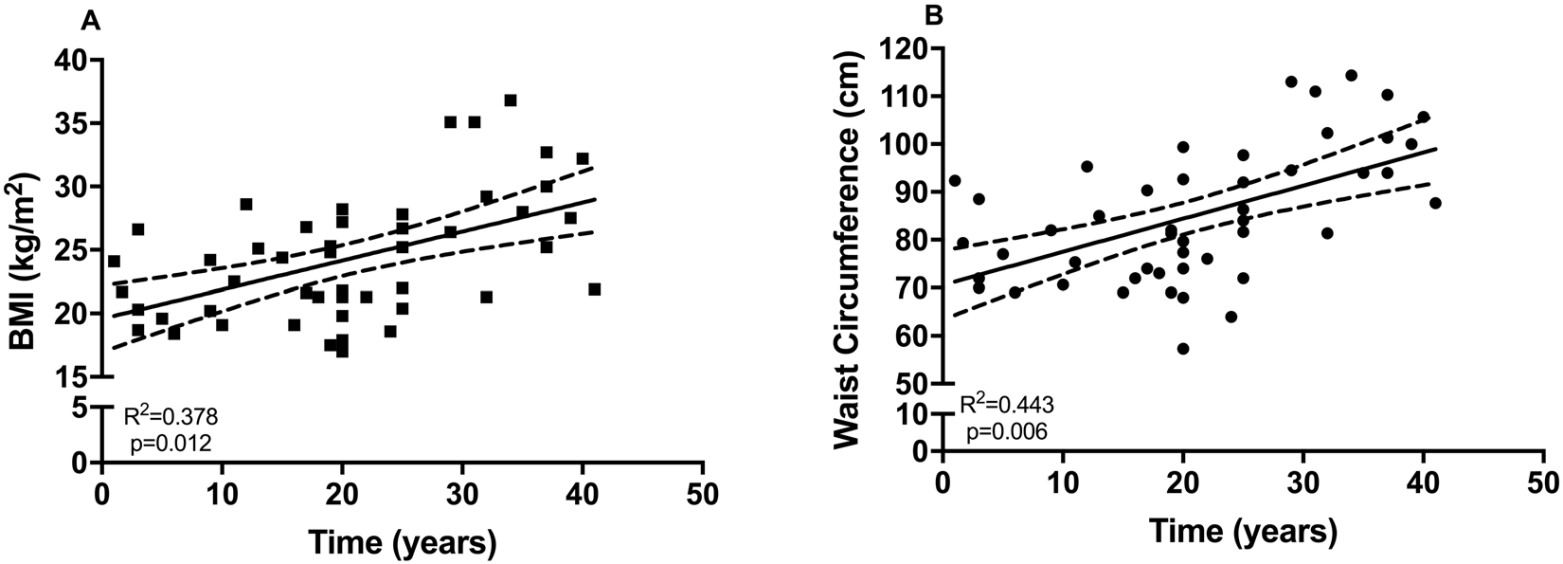
The association between length of stay in urban area with adiposity. The association between length of time in urban area with (**A**) body mass index (BMI) and (**B**) waist circumference are presented in scatter plot graphs (n = 49), and analysed using age-adjusted linear regression. The dotted line represents 95% confidence intervals. Each year increase of a time spent in urban area was associated with a significant increase in both (a) BMI [0.152 (0.036–0.269) kg/m^2^, p = 0.012] and (b) Waist Circumference [0.449 (0.135–0.762) cm, p = 0.006].

The mean length of stay of urban subjects in Jakarta was 20.7 (range: 1–40) years. The differences in metabolic profile between subjects living in rural and urban environments are summarized in Table 1. Urban subjects had a significantly higher homeostatic model assessment (HOMA) -IR compared to rural subjects [1.45 (1.06–1.90) vs 0.96 (0.80–1.13), respectively, p = 0.01]. Similarly, other metabolic parameters, such as 2 hour-blood-glucose, hemoglobin A1c (HbA1c), body mass index (BMI), waist circumference, and leptin level were significantly higher in urban subjects (Table 1). Interestingly, independent of age, increasing length of stay in urban area (in years) was positively associated with increasing BMI (in kg/m^2^) [estimate (95% CI), 0.15 (0.04–0.27), p = 0.01, Fig. 1A], waist circumference (in cm) [0.45 (0.14–0.76), p = 0.006, Fig. 1B], but not HOMA-IR [0.005 (−0.003–0.013), p = 0.18]. Increasing length of stay in urban area was also associated with a trend of increase in leptin level (in ng/mL) [0.013 (−0.001–0.027), p = 0.07].

**Table 1:** Comparison of metabolic profiles between subjects living in urban and rural area.

	Urban (n = 49)	Rural (n = 105)
Duration in urban (in years)	20.7 (1.0-40.0)	—
Age (in years)	39.3 (13.5)	44.5 (12.2)*
HOMA-IR	1.45 (1.06–1.90)	0.96 (0.80–1.13)*
Fasting Insulin (mU/L)	4.9 (3.8–6.4)	3.1 (2.5–3.8)**
Fasting Blood Glucose (mmol/L)	5.7 (1.4)	5.4 (0.9)
2h-Blood Glucose (mmol/L)	7.7 (3.2)	5.9 (1.9)**
HbA1c^#^ (mmol/L)	37.9 (14.3)	32.3 (6.6)*
HbA1c^#^ (%)	5.6 (1.3)	5.1 (0.6)*
Body Mass Index (kg/m^2^)	24.3 (4.9)	22.7 (4.0)*
Waist Circumference (cm)	84.9 (13.8)	79.3 (11.9)*
Adiponectin (µg/mL)	4.38 (3.31–5.78)	3.54 (3.09–4.07)
Leptin (ng/mL)	5.62 (3.98–7.92)	2.64 (2.06–3.38)*
CRP (mg/L)	1.57 (1.17–2.05)	1.67 (1.29–2.11)
Total IgE (IU/mL)	168 (105–271)	931 (702–1,235)**
Prevalence of STH (%, n/N)	5 (2/42)	57 (52/92)**

All variables are presented as mean and its standard deviation, however, HOMA-IR, fasting insulin, adiponectin, leptin, CRP, and total IgE level are presented as geomean (95%CI) and were log transformed for analysis, while duration in urban is presented as mean (range). Analysis for the difference between urban and rural group was performed using independent t-test (*p < 0.05, **p < 0.0001) ^#^Hba1c measurements were available in 42 and 95 of urban and rural subjects respectively. Abbreviation: HOMA-IR = the homeostatic model assessment of insulin resistance, CRP = C-reactive protein, STH = soil-transmitted helminth.

The prevalence of soil-transmitted helminth (STH) was significantly lower in the urban compared to rural subjects [5% (2/42) vs 57% (52/92), respectively, p < 0.0001]. Similarly, the levels of total IgE, often driven by STH infections^31^, were lower in the urban compared to rural subjects (168 (105–271) IU/mL vs 931 (702–1,235) IU/mL, respectively, p < 0.0001) (Table 1). As the number of subjects with current STH infections in urban area was very low (n = 2), it was not possible to assess the contribution of current STH infections to the HOMA-IR difference between urban and rural subjects. Therefore, we used the total IgE level as a proxy for past and current exposure to STH. The age-adjusted difference in HOMA-IR between urban and rural subjects was slightly attenuated [from estimated mean differences (95% CI), 0.09 (0.02–0.17), p = 0.001 to 0.08 (−0.00–0.17), p = 0.06] after further adjustment for total IgE level (Table 2). Moreover, adjustment for total IgE level also attenuated the age-adjusted difference in waist circumference [from 7.2 (2.0–11.3) cm, p = 0.001 to 4.2 (−0.5–8.8) cm, p = 0.08] and leptin level [from 0.36 (0.18–0.55) ng/mL, p < 0.0001 to 0.10 (−0.03–0.24) ng/mL, p = 0.14] (Table 2). When assessing the contribution of adiposity and leptin levels to the difference in HOMA-IR between urban and rural subjects, we observed that adjustment for waist circumference [to 0.02 (−0.04–0.08), p = 0.55] or both waist circumference and leptin level [to 0.01 (−0.06–0.07), p = 0.77] strongly attenuated the difference in HOMA-IR (Table 2).

**Table 2:** Associations between living in urban and rural area with HOMA-IR, leptin, and waist circumference.

Variables	Differences for each variable between urban and rural (rural group as the reference group)*					
	Crude	Model 1 (Age)	Model 2 (Age + Total IgE)	Model 3 (Age + Waist)	Model 4 (Age + Total IgE + Waist)	Model 5 (Age + Waist + Leptin)
HOMA-IR^$^	0.10 (0.02–0.17), p = 0.010	0.09 (0.02–0.17), p = 0.016	0.08 (−0.00–0.17), p = 0.061	0.02 (−0.04–0.08), p = 0.545	0.04 (−0.03–0.11), p = 0.294	0.01 (−0.06–0.07), p = 0.774
Leptin (ng/mL)^$^	0.33 (0.14–0.51), p = 0.001	0.36 (0.18–0.55), p < 0.0001	0.10 (−0.03–0.24), p = 0.137	0.11 (−0.01–0.22), p = 0.076	0.08 (−0.05–0.21), p = 0.216	—
Waist Circumference (cm)	5.6 (1.3–9.9), p = 0.010	7.2 (3.0–11.3), p = 0.001	4.2 (−0.5–8.8), p = 0.077	—	—	—

*Beta coefficient (95% CI) from linear regression. ^$^HOMA-IR and leptin level were log transformed for analysis. Model 1: adjusted for age. Model 2: adjusted for model 1 plus total IgE level. Model 3: adjusted for model 1 plus waist circumference. Model 4: adjusted for model 2 plus waist circumference. Model 5: adjusted for model 3 plus leptin level. Abbreviation: HOMA-IR = the homeostatic model assessment of insulin resistance.

In addition, we stratified rural and urban subjects based on STH infection status into three groups, resulting in an urban group without STH infections, a rural group without STH infections, and a rural group with STH infections. The highest mean level of HOMA-IR, waist circumference, and leptin was observed in the urban group without STH infections, followed by the rural group without STH infections and the lowest among the rural group with STH infections (Figure S1). The opposite relationship was observed for total IgE level (Figure S1).

### Comparison of metabolic responses after a short-term HFD intervention between subjects living in an urban and rural area

Among subjects who were included in the interventional part of the study (n = 34), we observed no significant differences between the age-and-BMI-matched urban (n = 17) and rural group (n = 17) in terms of HOMA-IR, adipose-IR index, CRP, and lipid levels at D-0 (Pre HFD). At this time point, serum CETP levels were significantly lower in the urban group [1.96 (0.58) µg/mL vs 2.59 (0.64) µg/mL, in urban and rural group respectively, p = 0.006]. Both groups showed a good compliance in terms of dietary intervention, all participants consumed all the cream provided and maintained their regular diet, resulting in a mean daily calorie intake that was ~60% higher compared to their regular diet, and ~56% of energy was derived from fat. The details of the dietary composition are shown in the Supplementary Table S1.

**Figure 2:**
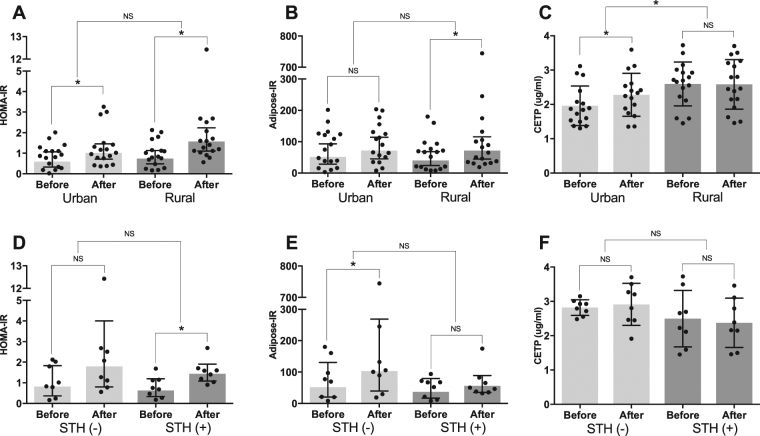
Comparison of Metabolic Responses to High-Fat Diet. HOMA-IR and adipose-IR index are presented as geometric mean and its corresponding 95% confidence interval, while CETP levels are presented as mean with its standard deviation. There were no significant differences in the increase of HOMA-IR (**A**), adipose-IR index (**B**) between urban and rural group, however, the increase in CETP level (**C**) was higher in the urban group. Furthermore, in rural group, there were no significant differences in the increase of HOMA-IR (**D**), adipose-IR index (**E**), and CETP level (**F**) between STH-infected and uninfected group. The difference between before and after intervention for each group was analysed using paired t-test, while the difference in the magnitude of changes for each parameter was analysed using linear mixed model (*p < 0.05, NS: p > 0.05).

Intervention with a 5-day HFD resulted in a significant increase of HOMA-IR in both the urban [from 0.78 (0.51–1.09) to 1.13 (0.78–1.57), p = 0.03] and rural group [from 0.87 (0.59–1.21) to 1.69 (1.01–2.45), p = 0.001] (Fig. 2A, Table S1**)**, which was mainly driven by the increase in fasting insulin level in both urban [from 4.05 (2.98–5.52) mU/L to 5.59 (4.18–7.47) mU/L, p = 0.02] and rural group [from 4.63 (3.42–6.26) mU/L to 7.68 (5.70–10.34 mU/L), p = 0.001] (Table S1). Comparing the changes in IR before and after intervention between urban and rural group, we observed no significant differences for either HOMA-IR [estimated mean differences (95% CI), −0.77 (−1.95–0.41), p = 0.21] (Fig. 2A, Table S2) or adipose-IR index [−41.2 (−115.1–32.7), p = 0.28] (Fig. 2B, Table S2).

Interestingly, we observed a significant increase in CETP levels after HFD intervention in the urban group only [from 1.96 (0.58) µg/mL to 2.28 (0.63) µg/mL, p = 0.004 in urban group vs from 2.59 (0.64) µg/mL to 2.58 (0.72) µg/mL, p = 0.93 in rural group) (Fig. 2C). Therefore, in comparison to the rural group, the increase in CETP level was significantly higher in urban group [0.33 (0.06–0.60) µg/mL, p = 0.02] (Fig. 2C, Table S2). However, as indicated above, the CETP levels were already much higher in the rural group at D-0 (Pre HFD), even higher than the D-6 (post-HFD) CETP level in the urban group. Intervention with the HFD did not significantly increase CRP levels in the two groups (Table S2). When assessing the effects of HFD on lipid levels, we observed no significant difference in changes in total cholesterol (TC), low-density lipoprotein cholesterol (LDL-C), and triglyceride (TG) levels between the urban and rural group, while the increase in high-density lipoprotein cholesterol (HDL-C) after intervention was significantly higher in the urban group compared to the rural group [0.09 (0.01–0.17) mmol/L, p = 0.04] (Table S2).

### The effect of current STH infections on the metabolic responses upon short-term HFD intervention

Next, due to the very low prevalence of STH infections in the urban group [6% (1/17)], the effect of current STH infections on the metabolic response towards a short-term HFD intervention was only assessed in the rural group of which 50% were positive for STH infection (8/16). Thus, our study was underpowered (power of 56%) to detect any differences in metabolic responses between STH-infected and uninfected subjects.

Despite a significantly lower baseline body weight in STH-infected subjects in comparison to STH-uninfected subjects [51.1 (11.0) kg vs 63.3 (10.2) kg, p = 0.04], there was no significant difference in the magnitude of increase in HOMA-IR [−1.08 (−3.38–1.22), p = 0.36] (Fig. 2D), adipose-IR [−87.8 (−222.1–46.4), p = 0.21] (Fig. 2E), or CETP level [−0.21 (−0.62–0.20) µg/mL, p = 0.32] (Fig. 2F) after HFD intervention. Interestingly, we observed a significantly higher increase in LDL-C level [0.27 (0.05–0.49) mmol/L, p = 0.03] after HFD intervention among STH-infected subjects in comparison to STH-uninfected subjects (Table S3). However, the level of LDL-C was much lower in the STH-infected group at D-0 in comparison to the STH-uninfected group [2.22 (0.31) mmol/L vs 2.97 (0.63) mmol/L, p = 0.01], and the level of LDL-C at D-6 in STH-infected group [2.44 (0.28) mmol/L] did not reach the level of LDL-C in the STH-uninfected group at D-0 (Table S3).

## Discussion

Our study showed that, in comparison to individuals living in a rural area, those living in an urban area had higher whole-body IR, as assessed by HOMA-IR, adiposity, and leptin levels. Whereas the higher whole-body IR was mainly mediated by the higher adiposity and leptin levels observed in urban individuals, differences in exposures to STH infection between urban and rural individuals, might contribute to a small extent to the differences observed in whole-body IR, adiposity or leptin levels. Intervention with a short-term HFD increased whole-body IR in both the urban and rural group. In comparison to the rural group, the CETP level was lower in the urban group, and the HFD intervention induced a stronger increase in CETP levels in this group. The presence of STH infections did not seem to have a protective effect on the acute induction of IR from short-term HFD. However, it has to be noted that our study was underpowered to detect an effect of STH.

Our study found that the higher whole-body IR in individuals living in urban area was mediated by the higher adiposity, as well as a higher leptin level, a pro-inflammatory adipokine, which has been previously reported to be associated with glucose metabolism^8,32^. The increase of adiposity and, to a lesser extent, leptin level, was positively associated with the duration of time spent in the urban environment. This suggests that a higher degree of acculturation in terms of urban lifestyle, drifting away from their traditional lifestyle^11^, could lead to a positive energy balance^20^, hence increasing adiposity over time. In addition, reduced exposures to environmental factors, such as to STH infections, which have been shown to have beneficial metabolic effects^13^ partly through the induction of type-2 and regulatory immune response^18,19^, might contribute to the difference in whole-body-IR, adiposity, and leptin level between urban and rural individuals. This was supported by our finding that the difference in whole-body IR, adiposity, and leptin level between urban and rural individuals was attenuated, but only slightly, after adjustment for total IgE level, a general marker for type-2 immune responses, and a proxy for past and current STH exposures^31^.

As expected, the overall metabolic profile of individuals living in a rural area, in term of adiposity and whole-body IR, was better in comparison to those living in an urban area. However, in contrast to our hypothesis, a short-term 5-day HFD intervention induced a similar increase of IR in both urban and rural individuals. As both groups were BMI-matched, these findings suggest that the direct protective metabolic effect of a combined past and current environmental exposures to helminths^13^, independent of their effect on adiposity, might be relatively weak in comparison to the strong induction of IR by the HFD intervention. Indeed, our group has recently reported that the increased IR in STH-infected subjects after deworming was mainly mediated by the increased adiposity^16^. Thus, adjusting for adiposity, in a way, remove the possible main pathway for STH-associated protection against the development for IR.

Although our study was underpowered to assess the effect of current STH infection, it is possible that the presence of current STH infections might not be sufficient to protect against a strong induction of IR by short-term HFD, as in rural subjects, the increase in IR after HFD in STH-infected and STH-uninfected subjects was similar. However, it is also possible that the HFD intervention in STH-infected subjects with lower body weight would have a stronger impact than in STH-uninfected subjects, thereby masking any protective effects of STH infections.

Interestingly, we observed that the baseline serum CETP level was significantly lower in urban subjects. As CETP is mainly produced by KCs, higher CETP level may represent an increase in hepatic macrophage (KC) content, hence liver inflammation^30^. Also, environmental factors in the rural area, mainly exposure to various infectious agents, such as microorganisms and parasites, may explain the increased CETP level. For instance, it has been shown that subjects with chronic hepatitis C virus infection have elevated serum CETP levels^33^. Supporting this, the prevalence of hepatitis in our rural study area was higher than our urban study area (4.3% vs 0.8%)^34^. However, currently, there are no available data connecting macrophage polarization status to CETP level and therefore further studies are needed^35^.

In contrast to what is seen in urban subjects^24,29^, we found no increase in CETP levels in rural subjects after the HFD intervention. It is possible that the lack of an increase in CETP levels in rural subjects was caused by the already high baseline CETP levels, thus precluding its further increase after HFD intervention.

Our results suggest an inflammation-independent mechanism of short-term HFD-associated induction of IR^23^ as there was no significant increase in CRP following HFD. Studies on the role of inflammation in HFD-associated induction of IR have shown conflicting results. In one study, an increase in CRP and expression of M1 macrophage markers in skeletal muscle was reported^24^, while in another, no increase was seen in circulating pro-inflammatory cytokines^36^.

In terms of lipid levels, while no significant changes in lipid levels were observed in rural group, HFD intervention significantly increased HDL-C level in urban group. Our study and other observed that urban subjects had a relatively higher fat intake than rural subjects^20^ at baseline. Thus, the relative difference in the changes of dietary composition before and after intervention^37–39^ between urban and rural individuals might potentially contribute to the difference in HDL-C level changes after intervention. In the rural group, we observed no significant changes in LDL-C in STH-uninfected subjects, whereas the HFD intervention resulted in a significant increase in LDL-C in STH-infected subjects. This might be related to the lower baseline LDL-C level and body weight in STH-infected subjects.

Our study is the first to compare the metabolic profile between people with the same genetic background, living in different environments (urban and rural) and to assess the metabolic responses to an intervention with a standardized short-term HFD. However, our study has several limitations. First, our study was only performed in male subjects, and potential differences in the outcomes might be observed in females. Next, due to the low prevalence of STH in urban area, our study could only assess the effect of current STH infections on HFD-induced IR in rural subjects. We also used a calculated HOMA-IR instead of the gold standard glycemic clamp to assess IR. In addition, there was no data available on physical activity, there were no biopsies of specific metabolic tissues (liver, muscle, adipose tissue), and we did not analyse the gut microbiota, all known to play an important role in metabolic profile and response.

In conclusion, in comparison to their rural ethnic counterparts, individuals living in an urban area had a higher whole-body IR, which was mainly mediated by their higher adiposity. The differences between urban and rural individuals in terms of past and current exposures to STH seem to have a relatively small contribution to the difference in whole-body IR. Contrary to our hypothesis, intervention with a short-term HFD induced similar increase in IR, in urban and rural individuals, and in helminth infected and uninfected subjects. However, well-powered larger studies are needed to determine which factors in terms of urbanization contribute to IR.

## Methods

### Study Design and Population

The present study consisted of a cross-sectional and an interventional study. The cross-sectional study was performed in an urban (Jakarta) and a rural area (Nangapanda, Ende, Flores island) in Indonesia. We recruited 49 males (18–65 years old) with Floresian ethnic background who had migrated from Flores island and lived in Jakarta for more than 1 year (urban group). As their rural counterparts, we recruited 105 Floresian males with a similar age range, randomly selected from three villages in Nangapanda with age stratification, as described previously^40^.

For the HFD intervention study, 17 from urban and 17 from rural area, age-and-BMI-matched healthy young male volunteers (18–40 years old) were recruited via local healthcare workers who informed their community, in both Nangapanda and Jakarta, of the study. BMI-matching was performed to assess whether the difference between urban and rural in term of past or current exposure to STH infections affect the HFD-associated increase in IR, independent of adiposity. Exclusion criteria were T2D, recent body weight changes, intake of medication that could affect inflammation or IR.

The study was approved by the Medical Ethical Committee of the Faculty of Medicine, Universitas Indonesia (556/H2.F1/ETIK/2014) and performed in accordance with the principles of the revised Declaration of Helsinki. All volunteers gave written informed consent before participation.

#### Cross sectional Study

In the cross-sectional study, we invited all subjects to come to the Field Study Centre (FSC) in both rural and urban area to undergo clinical measurements and blood sample collections. Stool samples were also collected. All clinical measurements and blood sample collections were performed after an overnight fast. Anthropometric measurements of body weight, height, and waist circumference were performed. BMI was calculated as weight in kg divided by square of height in meter.

After collection of fasting blood samples, we performed an oral glucose tolerance test (OGTT), in which blood glucose levels were re-measured 2 hours after subjects were given 75 g glucose dissolved in 200 mL of water (2h-BG). In this cross sectional study, we calculated HOMA-IR, a well-validated measure of whole-body IR in humans (HOMA-IR = fasting serum insulin (mU/L) × fasting glucose (mmol/L)/22.5)^41^, as our primary outcome. We also measured HbA1c, fasting blood glucose (FBG), fasting insulin, 2h-BG, BMI, waist circumference, adiponectin, leptin, high-sensitive C-reactive protein (hsCRP), total IgE, and prevalence of STH as our secondary outcomes.

#### Intervention Study

Subjects were examined before and after a 5-day HFD intervention, consisting of the subject’s regular diet supplemented with 375 mL cream (Greenfields™ Whipping Cream, Greenfields Indonesia Ltd, Jakarta, Indonesia) per day [1,500 kcal/day, 83% fat (60% saturated fat), 17% protein, 0% carbohydrate]. After baseline measurements, each subject received three bottles of 125 mL cream per day for five consecutive days. Subjects were instructed to continue their regular diet, and to consume one bottle of cream after each meal (3 meals per day) to make sure they could adhere to their regular dietary habits.

Subjects were asked to keep a food diary before and during the HFD intervention to estimate normal dietary intake and to check for compliance and compensatory behavior. Dietary assessment, using a 24 hours food recall, was performed by a trained dietician. Compliance was further assessed by interviewing the subject and collecting the bottles every day. During the study, subjects were asked not to change lifestyle habits. Measurements of clinical parameters and blood drawing were done on the day before starting the HFD intervention (D-0) and one day after the fifth day of the HFD intervention (D-6).

In this intervention study, we had HOMA-IR as our primary outcome. As our secondary outcomes, we measured adipose-IR index, a measure of adipose tissue IR, which was calculated as the product of the fasting serum free fatty acid (FFA) and insulin (Adipose-IR index = FFA[mM] × Insulin [pM])^42,43^. In addition, we also measured hsCRP, CETP, and lipid levels [TC, HDL-C, TG, LDL-C]. Due to limited amount of sera after intervention, adiponectin and leptin level were measured only at baseline. All others measurements for the interventional study were performed pairwise (before and after intervention).

### Laboratory measurements

Fasting blood glucose and 2h-post-load glucose were measured in capillary blood using Breeze®2 glucose meters (Bayer Health Care LLC, Basel, Switzerland) in the FSC. All sera, plasma and whole blood samples from rural area were frozen at −20 °C in the FSC, and subsequently shipped and stored at −80 °C in Faculty of Medicine Universitas Indonesia (FKUI), Jakarta, Indonesia and Leiden University Medical Centre (LUMC), Leiden, The Netherlands. All sera, plasma and whole blood samples from urban area were directly transported from FSC (Jakarta) to be stored at −80 °C in FKUI, and subsequently shipped and stored at −80 °C at LUMC.

Serum insulin concentrations were determined by a solid-phase, enzyme-labeled chemiluminescent immunometric assay, while HbA1c was measured using a cation-exchange chromatography (IC)-based high performance liquid chromatogtaphy (HPLC) assay. A latex-enhanced immunoturbidimetric method was used to measure hsCRP. Assays of TC, HDL-C, and TG were based on enzymatic colorimetric methods. These measurements have been described previously^16^.

Plasma CETP levels were measured with enzyme-linked immunosorbent assays (ELISA) kits according to the manufacturer’s instructions (DAIICHI CETP ELISA, Daiichi, Tokyo, Japan). FFA were measured using ELISA kits according to the manufacturer’s instructions (abcam ab 65341 FFA Quantification Assay Kit, Cambridge, UK). Adiponectin and leptin were also measured by using ELISA commercial reagents (DuoSet ELISA R&D System Europe Ltd, Abingdon, UK). The inter- and intra-assay coefficients of variance (CV) of adiponectin were 3.1% and 7.0% respectively. While for leptin, the inter- and intra-assay CV were 2.2% and 3.2% respectively. The levels of total IgE, an important determinant of total IgE levels^31^, was measured using ELISA as described previously^44^, with the inter- and intra-assay CV of 5.8% and 3.0% respectively. The presence of STH [hookworm (*Necator americanus, Ancylostoma duodenale*), *Ascaris lumbricoides*, *Trichuris trichiura*, *Strongyloides stercoralis*] was assessed using PCR as described in detail elsewhere^44,45^.

### Statistical analysis

Normally distributed continuous variables were summarized as mean and standard deviation [mean (SD)], while non-normally distributed data were summarized as geometric mean and its 95% confidence interval [geomean (95% CI)]. Based on previous studies^14,25^, we aimed to recruit 45 subjects from each urban and rural area for the cross-sectional study, while for the interventional study we aimed to recruit 15 subjects from each group (see Supplementary Material).

**Figure 3:**
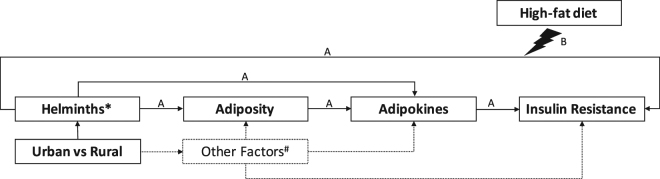
Conceptual framework. In the cross-sectional study (**A**), we assessed whether the differences in past or current exposure to helminths contribute to the difference in insulin resistance (IR) between subjects living in urban and rural area, and whether the observed difference in IR is independent from adiposity. In the high-fat diet (HFD) study (**B**), first, we assessed whether past or current exposure to helminths protect against the HFD-associated increase in IR, independent of adiposity. Next, we also assessed whether the presence of current helminth infection protect against the HFD-associated increase in IR. *Past and current exposure to helminths was assessed by measuring serum total IgE level, a general marker for Th2 responses, commonly induced by soil-transmitted helminth (STH). **Current exposure to helminths was assessed using stool PCR. ^#^Other factors that were not specifically assessed in this study.

The original plan for the linear regressions was based on a conceptual framework (Fig. 3) of the proposed causal pathways. In the cross-sectional study (**A**), we assessed whether the difference between urban and rural subjects, in term of past or current exposure to STH, by using total IgE level as a proxy, contributes to the difference in insulin resistance (IR) between subjects living in urban and rural area, and whether this difference in IR is independent from adiposity, by performing mediation analysis. Next, we further stratified the urban and rural group based on their STH infection status (see Supplementary Material). In addition, we also assessed the association between length of stay in urban area and metabolic profiles (IR, adiposity, and leptin) among subjects living in urban area using age-adjusted linear regression model.

In the HFD intervention study (**B**), first, we used mixed model to assess whether the difference between urban and rural in term of past or current exposure to STH infections affect the HFD-associated increase in IR, independent of adiposity, by matching both groups for BMI. Next, among subjects living in rural area, similar model was used to further assess whether the presence of current STH infections protect against the HFD-associated increase in IR. The mixed model analysis was performed using R software (lme4).

## Electronic supplementary material

Supplementary Material
