# Supplementary material for: Impact of rural-urban environment on metabolic profile and response to a 5-day high-fat diet
Source: Sci Rep. 2018 May 25;8:8149. doi: 10.1038/s41598-018-25092-6 (PMC5970191; doi:10.1038/s41598-018-25092-6)
Supplement: Supplementary file 1 — Supplementary Material [file 41598_2018_25092_MOESM1_ESM.docx]

**SUPPLEMENTARY MATERIAL**

**Impact of rural-urban environment**

**on metabolic profile and response to a 5-day high-fat diet**

Dicky L Tahapary^1,2,3,4#^, Karin de Ruiter^2#^, Farid Kurniawan^1,4#^, Yenny Djuardi^3,5^, Yanan Wang^6^, Siti M.E. Nurdin^7^, Elisa Iskandar^3,5^, Dominggus Minggu^8^, Em Yunir^1,4^, Bruno Guigas^2^, Taniawati Supali^3,5^, Patrick C.N. Rensen^6^, Erliyani Sartono^2^, Pradana Soewondo^1,4¤^, Dante S Harbuwono^1,4¤^, Johannes WA Smit^4,9¤^, Maria Yazdanbakhsh^2¤$^

1. Department of Internal Medicine, Division of Endocrinology, Dr. Cipto Mangunkusumo National General Hospital, Faculty of Medicine Universitas Indonesia, Jakarta, Indonesia.
2. Department of Parasitology, Leiden University Medical Center, Leiden, The Netherlands.
3. Nangapanda Community Research Cluster, The Indonesian Medical Education and Research Institute, Jakarta, Universitas Indonesia, Indonesia
4. Metabolic, Cardiovascular and Aging Research Cluster, The Indonesian Medical Education and Research Institute, Universitas Indonesia, Jakarta, Indonesia
5. Department of Parasitology, Faculty of Medicine Universitas Indonesia, Jakarta, Indonesia.
6. Department of Medicine, Division of Endocrinology, Leiden University Medical Center, Leiden, The Netherlands.
7. Laboratory Unit, South East Asian Minister of Education Organization Regional Centre For Food And Nutrition, Jakarta, Indonesia
8. Dr. W.Z. Johannes Hospital, Kupang, Indonesia
9. Department of Internal Medicine, Radboud University Medical Centre, Nijmegen, The Netherlands.

^#¤^These authors have contributed equally, ^$^Corresponding author

**Figure S1. Comparison of metabolic profile between urban participants and rural participants (rural participants stratified by helminth infection status)**

The levels of total IgE, HOMA-IR, waist circumference, and leptin on different group of living area and soil-transmitted helminth (STH) infection status are presented as geometric mean and its 95% confidence interval, except for waist circumference which are presented as mean and its standard deviation. The number of urban subjects with helminth infections was very low (2/42) and was not included in this graph. Trend analysis was performed between three groups, namely: (1) rural subjects with STH infections [Rural (+)], (2) rural subjects without STH infections [Rural (-)], and (3) urban subjects without STH infections [Urban (-)]. Total IgE level was the lowest in Urban (-) group and progressively become higher in Rural (-) and Rural (+) groups (**A**). The contrary was observed for HOMA-IR (**B**), waist circumference (**C**), leptin level (**D**). *p<0.05 in unadjusted model, **p<0.05 in age-adjusted model, ***p<0.05 in age-waist circumference-adjusted model.

**Table S1. Comparison of dietary compositions between urban and rural participants**

|  | **Urban** | | **Rural** | |
| --- | --- | --- | --- | --- |
|  | **Pre-HFD*** | **HFD** | **Pre-HFD*** | **HFD** |
| Energy (kcal) | 1617.6 (78.2) | 2533.0 (124.0) | 1890.3 (433.7) | 2822.6 (248.6) |
| Fat (g) | 51.8 (23.4) | 161.2 (7.5) | 50.7 (25.1) | 173.9 (13.9) |
| Carbohydrate (g) | 222.1 (69.3) | 200.1 (49.6) | 268.9 (66.4) | 220.2 (32.3) |
| Protein (g) | 67.2 (9.1) | 68.8 (5.6) | 78.0 (24.3) | 81.5 (16.8) |
| Fiber (g) | 9.4 (3.9) | 9.5 (1.5) | 10.0 (3.1) | 11.3 (2.3) |

*At baseline, the proportion of energy derived from fat, carbohydrate, and protein for subjects living in urban area were 29%, 55%, and 16% respectively, while for subjects living in rural area the percentages were 24%, 58%, and 18% respectively.

**Table S2. Comparison of metabolic responses towards a short-term HFD between subjects living in an urban and rural area**

|  | **Urban n=17** | | | **Rural n=17** | | | **Comparison of the magnitude of changes between urban and rural subjects**** | |
| --- | --- | --- | --- | --- | --- | --- | --- | --- |
| **Variables** | **Pre HFD** | **Post HFD** | **p-value*** | **Pre HFD** | **Post HFD** | **p-value*** | **Estimated differences** | **p-value** |
| Age (years) | 30.1 (6.4) | - | **-** | 29.5 (8.0) | - | **-** | - | - |
| Body Mass Index (kg/m^2^) | 23.1 (4.7) | - | **-** | 21.6 (3.6) | - | **-** | - | - |
| HOMA-IR | 0.78 (0.51 – 1.09) | 1.13 (0.78 – 1.57) | **0.03** | 0.87 (0.59 – 1.21) | 1.69 (1.01 – 2.45) | **0.001** | -0.77 (-1.95 – 0.41) | 0.21 |
| Fasting Blood Glucose (mmol/L) | 5.15 (0.44) | 5.23 (0.50) | 0.59 | 4.96 (0.19) | 5.46 (0.73) | **0.005** | **-0.42 (-0.82 - -0.03)** | **0.04** |
| Fasting Insulin (mU/L) | 4.05 (2.98 – 5.52) | 5.59 (4.18 - 7.47) | **0.02** | 4.63 (3.42 – 6.26) | 7.68 (5.70 – 10.34) | **0.001** | -2.35 (-6.55 – 1.84) | 0.28 |
| Adipose-IR Index | 51.6 (28.5 – 93.3) | 71.9 (45.0 – 114.7) | 0.23 | 40.5 (24.0 – 68.4) | 72.0 (44.8 – 115.7) | **0.006** | -41.2 (-115.1 – 32.7) | 0.28 |
| Free Fatty Acid (mmol/L) | 3.93 (3.37 – 4.59) | 3.43 (2.91 – 4.04) | 0.29 | 2.83 (2.42 – 3.30) | 2.65 (2.27 – 3.09) | 0.39 | -0.32 (-1.24 – 0.59) | 0.49 |
| CETP (µg/mL)^#^ | 1.96 (0.58) | 2.28 (0.63) | **0.004** | 2.59 (0.64) | 2.58 (0.72) | 0.93 | **0.33 (0.06 – 0.60)** | **0.02** |
| CRP (mg/L) | 2.04 (0.94 – 4.74) | 2.24 (1.21 – 3.75) | 0.82 | 1.14 (0.59 – 1.89) | 1.06 (0.58 – 1.71) | 0.81 | -0.31 (-3.61 – 2.98) | 0.85 |
| Total Cholesterol (mmol/L) | 4.11 (0.61) | 4.13 (0.59) | 0.84 | 4.38 (0.61) | 4.42 (0.71) | 0.65 | -0.02 (-0.29 – 0.24) | 0.87 |
| Triglyceride (mmol/L) | 1.29 (0.53) | 1.31 (0.54) | 0.83 | 1.35 (0.36) | 1.24 (0.43) | 0.37 | 0.14 (-0.17 – 0.44) | 0.38 |
| HDL-C (mmol/L) | 1.08 (0.25) | 1.18 (0.23) | **0.01** | 1.19 (0.25) | 1.20 (0.24) | 0.60 | **0.09 (0.01 – 0.17)** | **0.04** |
| LDL-C (mmol/L) | 2.44 (0.59) | 2.35 (0.570 | 0.251 | 2.57 (0.61) | 2.65 (0.59) | 0.21 | -0.17 (-0.36 – 0.01) | 0.08 |

All variables are presented as mean and its standard deviation, however, HOMA-IR, Fasting Insulin, Adipose-IR Index, Free Fatty Acid, and CRP levels are presented as geomean (95%CI). *The differences between before and after HFD intervention were analysed using paired t-test. **The difference in changes (before and after HFD) of different parameters between urban and rural were analysed using linear mixed model and are presented as [Estimated Differences in Changes (95%CI), p-value]. ^#^CETP measurements were only available for 33 subjects. Abbreviation: HOMA-IR= homeostatic model assessment of insulin resistance, CETP= cholesteryl ester transfer protein, CRP= C-reactive protein, HDL-C= high-density lipoprotein cholesterol, LDL-C= low-density lipoprotein cholesterol.

**Table S3. Comparison of metabolic responses towards a short-term HFD between STH-infected and uninfected subjects living in rural area**

|  | **STH-infected n=8** | | | | **STH-uninfected n=8** | | | | **Comparison of the magnitude of changes between STH-infected and STH-uninfected subjects**** | |
| --- | --- | --- | --- | --- | --- | --- | --- | --- | --- | --- |
| **Variables** | Pre HFD | Post HFD | p-value^*^ | Pre HFD | | Post HFD | p-value^*^ | Estimated differences | | p-value |
| Age (years) | 27.0 (9.6) | - | **-** | 32.0 (6.3) | | - | **-** | - | | - |
| Body Mass Index (kg/m^2^) | 20.1 (3.5) | - | **-** | 23.1 (2.4) | | - | **-** | - | | - |
| HOMA-IR | 0.73 (0.37 – 1.19) | 1.47 (1.08 – 1.93) | **0.002** | 1.00 (0.45 – 1.75) | | 2.03 (0.72 – 4.34) | **0.06** | -1.08 (-3.38 – 1.22) | | 0.36 |
| Fasting Blood Glucose (mmol/L) | 5.03 (0.22) | 5.69 (0.88) | **0.04** | 4.90 (0.17) | | 5.26 (0.57) | 0.13 | 0.30 (-0.30 – 0.90) | | 0.34 |
| Fasting Insulin (mU/L) | 4.04 (2.60 – 6.29) | 6.80 (5.17 – 8.95) | **0.008** | 5.12 (2.89 – 9.08) | | 8.97 (4.69 – 17.17) | **0.04** | -3.74 (-11.67 – 4.18) | | 0.36 |
| Adipose-IR Index | 36.6 (16.9 – 79.6) | 55.8 (34.9 – 89.0) | 0.22 | 51.7 (20.4 – 73.4) | | 103.2 (39.6 – 268.8) | **0.02** | -87.8 (-222.1 – 46.4) | | 0.21 |
| Free Fatty Acid (mmol/L) | 2.88 (2.41 – 3.45) | 2.44 (1.94 – 3.09) | 0.21 | 3.03 (2.42 – 3.83) | | 2.99 (2.34 – 3.81) | 0.87 | -0.37 (-1.18 – 0.44) | | 0.37 |
| CETP (µg/mL) | 2.49 (0.82) | 2.38 (0.72) | 0.46 | 2.82 (0.23) | | 2.91 (0.61) | 0.59 | -0.21 (-0.62 – 0.20) | | 0.32 |
| CRP (mg/L) | 0.96 (0.28 – 2.00) | 1.60 (1.48 – 3.57) | 0.13 | 1.32 (0.29 – 3.17) | | 0.71 (0.39 – 1.11) | 0.25 | 2.27 (-0.63 – 5.18) | | 0.13 |
| Total Cholesterol (mmol/L) | 4.07 (0.17) | 4.23 (0.33) | 0.20 | 4.77 (0.68) | | 4.70 (0.90) | 0.69 | 0.23 (-0.12 – 0.58 | | 0.21 |
| Triglyceride (mmol/L) | 1.32 (0.28) | 1.14 (0.46) | 0.40 | 1.40 (0.46) | | 1.38 (0.41) | 0.92 | -0.16 (-0.67 – 0.35) | | 0.54 |
| HDL-C (mmol/L) | 1.24 (0.25) | 1.27 (0.28) | 0.50 | 1.17 (0.26) | | 1.16 (0.20) | 0.88 | 0.03 (-0.07 – 0.13) | | 0.54 |
| LDL-C (mmol/L) | 2.22 (0.31) | 2.44 (0.28) | **0.01** | 2.97 (0.63) | | 2.92 (0.74) | 0.65 | **0.27 (0.05 – 0.49)** | | **0.03** |

All variables are presented as mean and its standard deviation, however, HOMA-IR, Fasting Insulin, Adipose-IR Index, Free Fatty Acid, and CRP levels are presented as geomean (95%CI). *The differences between before and after HFD intervention were analysed using paired t-test. ** The difference in changes (before and after HFD) of different parameters between STH-infected and STH-uninfected were analysed using linear mixed model and are presented as [Estimated Differences in Changes (95%CI), p-value]. Abbreviation: HOMA-IR= homeostatic model assessment of insulin resistance, CETP= cholesteryl ester transfer protein, CRP= C-reactive protein, HDL-C= high-density lipoprotein cholesterol, LDL-C= low-density lipoprotein cholesterol.

**Supplementary Methods**

Statistical Analysis

In the cross-sectional study, sample size was calculated to aim at a difference in HOMA-IR between urban and rural group of 0.5. The SD of HOMA-IR from previous study was 0.84.^1^ We used a significance level of 5% and a power of 80%, thus we needed at least 45 subjects for each group. For the interventional study, sample size was calculated to aim at a difference in changes of HOMA-IR between urban and rural group of 0.70. The SD of the HOMA-IR changes after HFD intervention from previous study was 0.68.^2^ We used a significance level of 5% and a power of 80%, thus we needed at least 15 subjects per group or 30 subjects in total. Next, to assess STH effect on the metabolic response upon HFD intervention we used similar calculation, aiming at having at least 15 subjects per group.

For the cross-sectional study, we further stratified the urban and rural group based on their STH infection status. However, as the number of urban subjects with STH infections was very low and therefore was excluded from analysis, eventually we had three groups: rural subjects with STH infections, rural subjects without STH infections, and urban subjects without STH infections. We calculated variance inflation factors (VIFs) to check multicollinearity in our regression models and VIF values below 4 were considered appropriate. Due to multicollinearity between BMI and WC, we used WC as clinical marker for adiposity. Analyses were performed using IBM Statistics 23.

For the HFD intervention study, to compare the parameter before and after the HFD intervention for each group, whenever appropriate, paired t-test or Wilcoxon-signed ranked test was performed. A mixed model was applied to assess mean differences before and after intervention between group. Groups were modelled as fixed effects, and to model correlation within subjects, random-specific intercept was used.

References

1. Wiria, A. E. *et al.* Infection with Soil-Transmitted Helminths Is Associated with Increased Insulin Sensitivity. *PLoS One* **10**, e0127746, doi:10.1371/journal.pone.0127746 (2015).
2. Bakker, L. E. *et al.* A 5-day high-fat, high-calorie diet impairs insulin sensitivity in healthy, young South Asian men but not in Caucasian men. *Diabetes* **63**, 248-258, doi:10.2337/db13-0696 (2014).
